# Quantitative and qualitative analysis of the quality of life of Type 1 diabetes patients using insulin pumps and of those receiving multiple daily insulin injections

**DOI:** 10.1186/s12955-022-02029-2

**Published:** 2022-08-01

**Authors:** Lilian Tzivian, Jelizaveta Sokolovska, Anna E. Grike, Agate Kalcenaua, Abraham Seidmann, Arriel Benis, Martins Mednis, Ieva Danovska, Ugis Berzins, Arnolds Bogdanovs, Emil Syundyukov

**Affiliations:** 1 Faculty of Medicine, University of Latvia, Jelgavas Str. 3, Riga, Latvia; 2 Faculty of Humanities, University of Latvia, Riga, Latvia; 3 Faculty of Medicine, Riga Stardins University, Riga, Latvia; 4 Longenesis Ltd, Riga, Latvia; 5 Questrom Business School, Boston University, Boston, MA 02215 USA; 6 Digital Business Institute, Health Analytics and Digital Health, Boston University, Boston, MA 02215 USA; 7 Faculty of Industrial Engineering and Technology Management, Holon Institute of Technology, 5810201 Holon, Israel; 8 Faculty of Digital Technologies in Medicine, Holon Institute of Technology, 5810201 Holon, Israel; 9 Faculty of Computing, University of Latvia, Raina boulevard 19, Riga, 1050 Latvia

**Keywords:** Type 1 diabetes mellitus, Insulin pump, Multiple daily insulin injections, Quality of life, Real World Data digital tool, Diabetes-related expenses, Comparative effectiveness research, Health economics

## Abstract

**Introduction:**

Insulin pump therapy represents an alternative to multiple daily injections and can improve glycemic control and quality of life (QoL) in Type 1 diabetes mellitus (T1DM) patients. We aimed to explore the differences and factors related to the T1DM-specific QoL of such patients in Latvia.

**Design and methods:**

A mixed-method cross-sectional study on 87 adult T1DM patients included 20 pump users and 67 users of injections who participated in the quantitative part of the study; 8 pump users and 13 injection users participated in the qualitative part. Patients were invited to participate using a dedicated digital platform. Their QoL and self-management habits were assessed using specially developed questionnaires adapted to Latvian conditions. Multiple logistic regression models were built to investigate the association between social and self-management factors and patients’ QoL. In addition, qualitative analysis of answers was performed.

**Results:**

Insulin pump users were younger, had higher incomes, and reported higher T1DM expenses than users of multiple daily injections. There were no differences in self-management between the groups; Total QoL differed at the 0.1 significance level. In fully adjusted multiple logistic regression models, the most important factor that increased Total QoL was lower T1DM-related expenses (odds ratio, OR 7.02 [95% confidence interval 1.29; 38.0]). Men and those with more years of living with T1DM had better QoL (OR 9.62 [2.20; 42.1] and OR 1.16 [1.05; 1.29], respectively), but the method of administration was not significantly associated with QoL (OR 7.38 [0.87; 62.9]). Qualitative data supported the results of quantitative analysis.

**Conclusions:**

QoL was the main reason to use an insulin pump, while the expense was the main reason to avoid the use of it or to stop using it. Reimbursement policies thus should be considered to enable patients to choose the more convenient method for themselves.

**Supplementary Information:**

The online version contains supplementary material available at 10.1186/s12955-022-02029-2.

## Introduction

Type 1 diabetes mellitus (T1DM) is a chronic autoimmune characterized by hyperglycemia due to loss of insulin producing cells of the pancreas that can end in diabetic coma and eventually death [1, 2]. T1DM incidence has increased on average 3–4% over the past 30 years [3], reaching an incidence of 15 people per 100,000 and a prevalence of 9.5 per 10,000 worldwide [4]. In Latvia, there were 4169 patients with T1DM in 2015 (prevalence of 211.7 per 100,000), and an incidence of 13.5 people per 100,000 [5].

The main therapy for T1DM patients is insulin regulation via multiple daily injections or continuous subcutaneous infusions using an insulin pump. Patients aim for glycated hemoglobin (HbA1c) levels below 7% [1] without an unacceptable incidence of hypoglycemia [6]. This process demands a certain amount of self-management, such as treatment diaries and recording and interpretation of blood sugar levels. Some patients, however, struggle with these tasks and fail to successfully continue a therapy, especially in the case of multiple daily injections. The use of an insulin pump as a technological solution can simplify efforts to manage the process and to maintain desired levels of blood glucose [7].

Administration of insulin via a pump improves glycemic control with fewer hypoglycemic episodes in T1DM subjects previously conventionally treated with multiple daily injections, achieving a significant reduction in HbA1c. Meta-analyses reveal that in patients treated with an insulin pump, Hb1A1c decreased more pronouncedly and reported insulin requirements were lower [8] than for injection patients, especially young children. Severe hypoglycemia episodes were rare, indicating better glycemic control and lower incidence of nocturnal hypoglycemia [8–10].

The quality of life (QoL) of patients with T1DM is affected by complications and fear of them and is lower than that of healthy peers [11]. Using an insulin pump reduces fear of severe hyperglycemia and diabetic coma [12, 13]. Patients using a pump have more flexible possibilities regarding meals, diet, everyday activities, and socialization [14], as the pump supports improved self-management habits [15–17]. Some additional non-health-related benefits, such as reduced worry about supplies while traveling, can significantly improve patients’ QoL as well [12]. However, the pump itself and related physical restrictions can be mentioned as disadvantages [11, 14]. The most prominent problem with an insulin pump is the expense. There is a large difference in cost between injections and a pump. Although studies show that there is a good value for money in the use of a pump, many adult patients may be unable to afford one [12].

According to the Latvian Diabetes Association, 3700 patients in Latvia currently have T1DM [18]. Insulin pumps are covered by the state until 18 years old [19], but adult patients must pay for the pump itself (approximatively 3500 EUR) and also cover the cost of pump-related disposables, amounting to more than 100 EUR per month. Considering the low-income level in Latvia (an average of 583 EUR per household member per month, in 2020) [20], insulin pump therapy is a huge financial burden. In these circumstances, the investigation of factors related to the QoL of patients using different methods of insulin administration can identify appropriate changes to reimbursement policies to improve such patients’ disease-related conditions.

The aim of this mixed-method cross-sectional study was to compare the QoL and T1DM-related self-management of two groups of patients residing in Latvia—insulin pump users and those who use multiple daily insulin injections, and to investigate factors associated with their QoL. Our main hypotheses were as follows:

1. The QoL of insulin pump users is better than that of injection users, and T1DM-related self-management is easier for pump users than injection users.
2. Easier T1DM-related self-management is associated with better QoL.

We investigated also specific reasons to use or not the pump or injections, including the reasons for changes between different methods of insulin administration, using both qualitative and quantitative methodologies. Our main hypothesis was that the major reason for using a specific method of administration and for changes in the method used is treatment-related expenses.

## Research design and methods

### Study design and population

The mixed-method cross-sectional study was conducted in April and May 2021 and consisted of a quantitative part and a qualitative part. We chose a combined approach due to the small number of insulin users in Latvia that lead to imprecision in the quantitative results. All T1DM patients at least 18 years of age who signed informed consent forms were eligible to participate in the study. As the total number of adult insulin pump users in Latvia is very small (about 40 users to our knowledge), we invited all of them to participate in the quantitative part of this study. The number of multiple injection users was planned to be in a proportion of 1:2 according to the enrolled sample of insulin pump users, and the calculated power of the study in that case was 80%. For the qualitative part of the study, the number of participants depended on their agreement and on the saturation of interviews—a lack of new information collected during the additional interviews. The saturation was defined by the investigator during the interviewing process. Both groups of participants—those using insulin pumps and those receiving multiple insulin injections—were enrolled in the qualitative part of the study. The study was approved by the Scientific Research Ethic Commission of the Institute of Cardiology and Regenerative Medicine of the University of Latvia on February 2, 2021.

### Methods of enrolment of the study participants

**Fig. 1:**
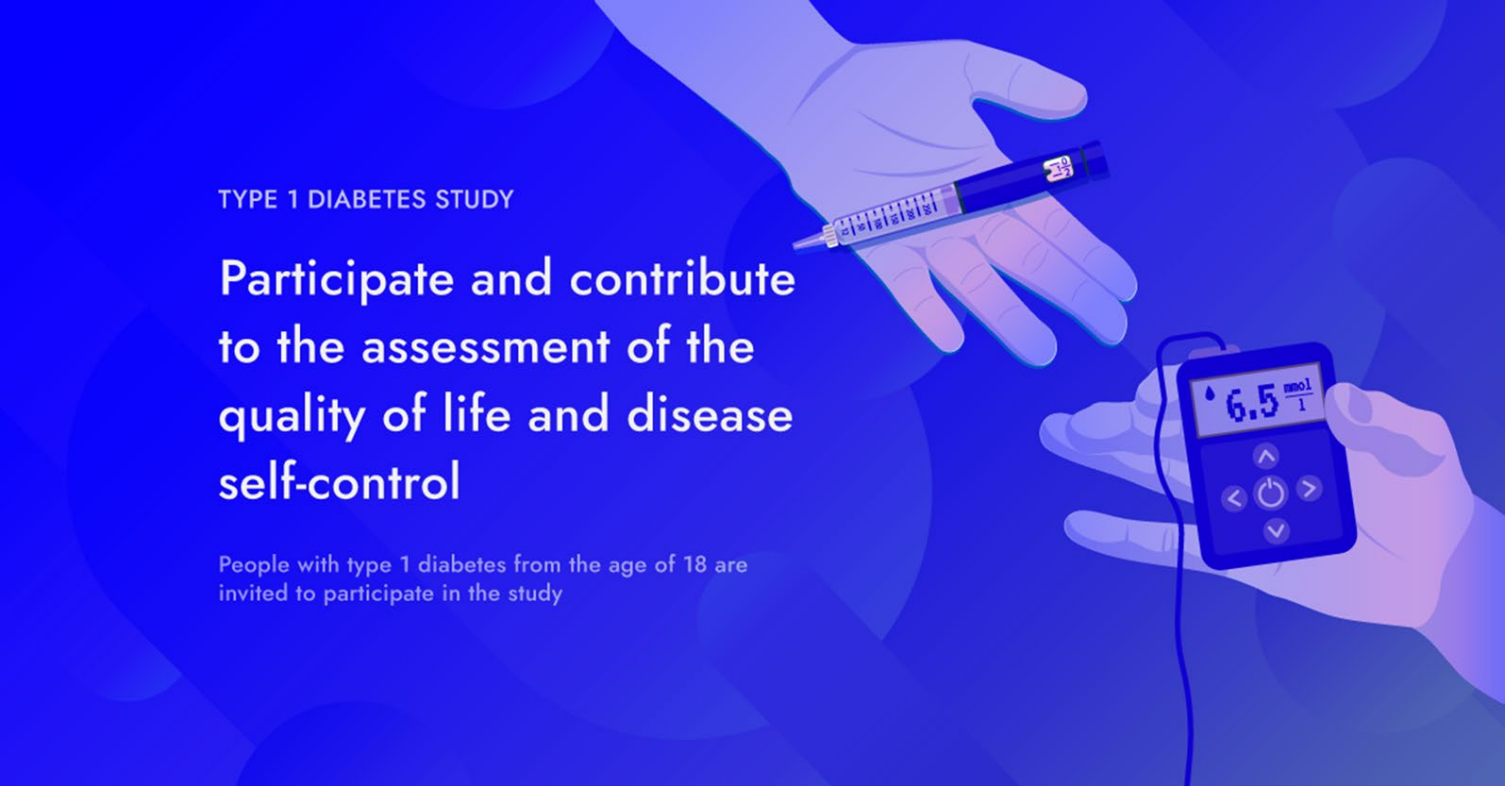
Visual engagement material used in study invitations

Patients learned about the study from e-mail materials received from doctors, patient organizations’ representatives, or diabetes nurses. Additionally, they could learn about the study from posts in the closed Facebook group “Diabetes in Latvia”. We also identified potential study participants using metadata from the longitudinal study “LatDiane: Latvian diabetic nephropathy study”, initiated in 2013 [21]. Currently, more than 355 well-characterized patients with Type 1 diabetes are in the LatDiane study. Invitations included a description of the study and its objectives, as well as a technical guide for onboarding on the digital platform developed for this study [22]. Patients were invited to participate using a digital engagement platform, equipped with a dynamic e-consent management tool (Fig. 1). The web-based and mobile-ready engagement platform was developed as a collaboration among clinicians, epidemiologists, and data protection and digital health specialists. The website of this study includes detailed instructions in Latvian and Russian, conditions for participation, information regarding the aims and organization of the study, and a contact section. Participants were asked to provide their consent (which could be dynamically managed on the platform, e.g., for opt-out) for data processing, in compliance with the General Data Protection Regulation (GDPR) [23].

**Fig. 2:**
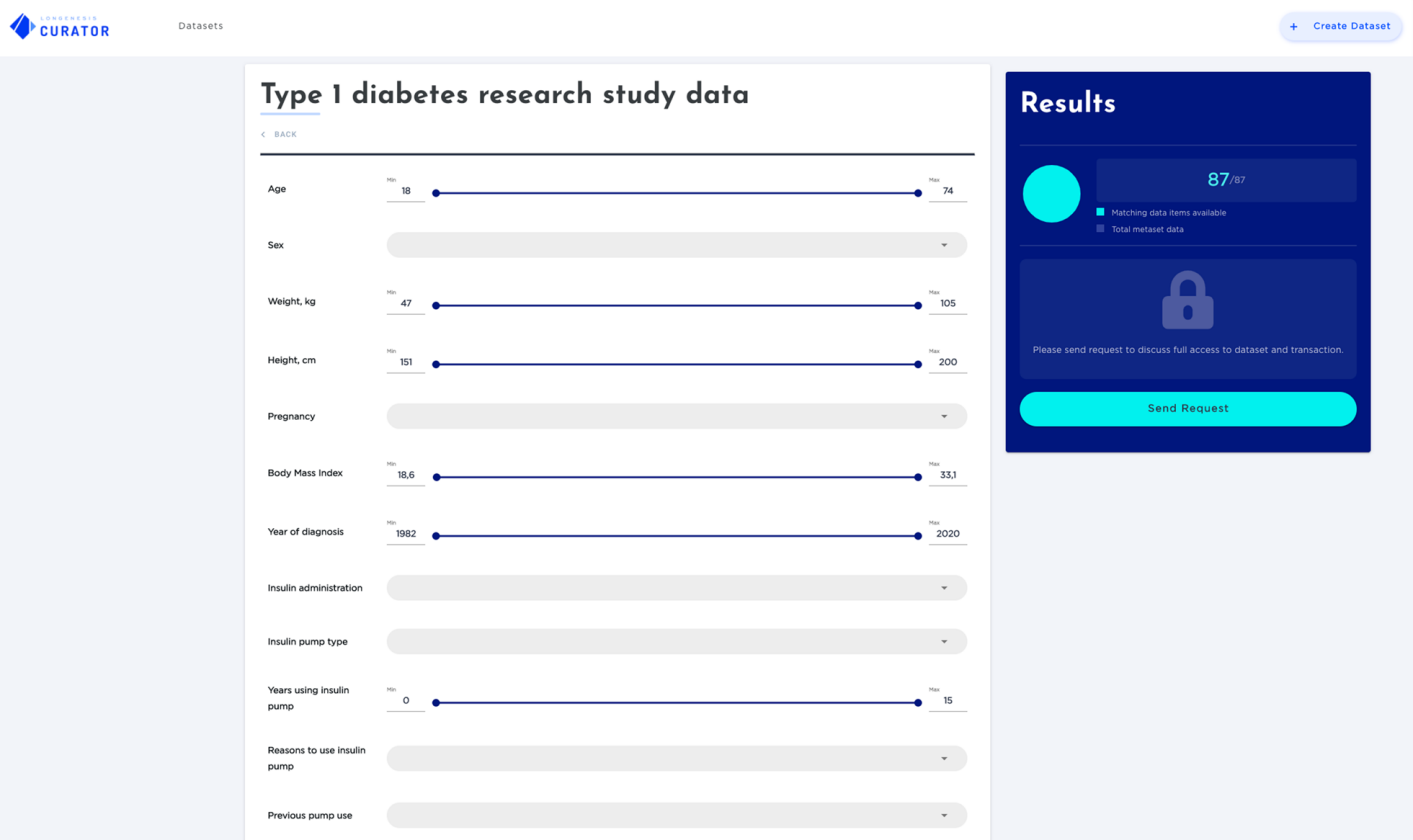
Electronic platform—research study metadata query view

Once they provided their informed consent, participants were invited to complete the online questionnaire and were informed about the time slots available for semi-structured interviews. After data collection, the system extrapolated a dataset that described the user survey input results, fully separated from the actual database (Fig. 2).

### The quantitative part of the study

The quantitative part of the study included self-reported socio-demographic information (age, gender, education, living conditions, financial needs, and income) and disease-related factors, including weight and height for calculation of the body-mass index (BMI) (Additional file 1: Supplement 1), years of living with T1DM, number of hypoglycemia incidents per week, number of hypoglycemia incidents per half-year, HbA1c in the last medical check, number of HbA1c checks during the year, and T1DM expenses (Additional file 1: Supplement 2).

We developed questionnaires for this study that consider conditions in Latvia. The QoL questionnaire comprised 35 questions divided into five blocks: Signs and symptoms (15 questions), Therapy (5 questions), Care (6 questions), Concerns (4 questions), and Communication (6 questions) (Additional file 1: Supplement 3). The self-management questionnaire consisted of 19 questions divided into three blocks: General, Diet, Physical activities (Additional file 1: Supplement 4).

All questionnaires were available in the two main languages in use in Latvia – Latvian and Russian. Translation and back translation of questionnaires were performed by two independent professional translators.

### Statistical analysis of the quantitative part

The reliability of the questionnaires was checked using the alpha-Cronbach’s test (α) after the first 20 participants had responded (α > 0.75 for all blocks of the questionnaire). These participants were subsequently included in the study sample, and their answers were analyzed together with those of other participants. For both questionnaires, we transformed the answers into values between 0 and 100 and then calculated means for each block. We further calculated the Total QoL and Total SM (self-management) scores as the means of all questions in their respective surveys. Higher values mean better QoL or better self-management.

We next compared pump users and injection users for all demographic variables, using central and dispersion measures according to the type of each variable. We used the Mann–Whitney test to compare qualitative variables and Chi-squared or Cramer’s V tests to compare the quantitative ones. We investigate the correlation between individual subscales and Total QoL and Total SM using Spearman correlation. We considered a two-sided 0.1 significance level for this stage of analysis.

We built multiple logistic regression models for Total QoL, dividing the Total QoL variable at the median (‘worse’ ≤ 67.9’, ‘better’ > 67.9). Variables found univariately statistically significantly related to Total QoL at the 0.1 significance level were included in logistic regression models together with demographic and T1DM-related variables that were found significantly different between pump users and injection users. The full adjustment set included the method of administration, age, sex, education, income, T1DM expenses, years with T1DM, and Total SM. We choose the best model fit according to the − 2 Log-likelihood test. *p* value < 0.05 was considered statistically significant for this part of the study. Odds ratio (ORs) and 95% confidence intervals were presented for multiple logistic regression models. We used Statistical Package for Social Science (SPSS) software (26th version) for the statistical analysis [24].

### Additional and sensitivity analyses

For additional insight, we asked pump users about the number of years they have used the pump and their reasons for using one (6 categories: QoL, insulin dosing, less pain, less hypoglycemia, just trying, and other). We asked injection users about reasons for not using a pump (6 categories: no trust, expensive, lack of appropriate model, lack of willingness, negative information, other) and reasons for ceasing to use a pump if it was used previously (5 categories: expensive, lack of trust, not comfortable, not resultative, and other). For the sensitivity analysis, we built multiple logistic regression models for two QoL blocks that significantly differed between the user groups (Therapy and Concerns), dividing the results for each block by the median value into ‘worse’ and ‘better’ and using the same set of covariates.

### Qualitative part of the study

The qualitative part of the study consisted of analysis of semi-structured interviews performed face-to-face or via telephone or video chats. Interviews were recorded, coded, transcribed according to their major theme, and analyzed using Nvivo software (version 12) [25] to obtain subcategories of each major theme. Coding of interviews included changing participants’ names. In this paper, we provide part of the results of the qualitative analysis as support for interpreting the quantitative results.

## Results

### Study participants

We enrolled 87 T1DM patients in the quantitative part of the study: 20 pump users and 67 injection users. Both groups included mostly women. Pump users generally had at least some secondary education and had higher incomes, while injection users mostly had just a high school education. Pump users were younger (mean age 21.5 years, standard deviation (SD) 4.4) than injection users (mean age 33.6 years, SD 11.0). The groups did not differ by other socio-demographic characteristics.

HbA1c values at the last medical check did not differ significantly between the groups. In both groups, most of the patients performed one medical check during 2020 and till May 2021. Only 10% of pump users and 15% of injection users mentioned four medical checks during this period. There were no differences between pump users and injection users in this parameter. T1DM-related expenses were statistically significantly higher for pump users: for 94.7% of them, these expenses were more than 100 EUR/month; just 29.2% of injection users had similarly high expenses (Table 1).

**Table 1:** Demographic and T1DM-related characteristics of participants, by method of insulin administration

Variable	Categories	Pump users, N = 20	Injection users, N = 67	*p *value
Age, median (25%–75%)		20.0 (18.2–22.0)	31.0 (25.0–40.0)	< 0.01
Female gender, N (%)		12 (60.0)	43 (64.2)	0.18
Pregnancy for female, N (%)	Not pregnant at the time of the study	8 (40.0)	24 (35.8)	0.62
Education	Higher education Less than higher education	6 (14.0) 14 (31.8)	37 (86.0) 30 (68.2)	0.07
Living conditions	Not alone Alone	18 (24.0) 2 (16.7)	57 (76.0) 10 (83.3)	0.73
BMI, median (25%–75%)		24.8 (21.6 – 29.4)	23.5 (20.8 – 26.5)	0.19
Income	≤ 600 Euro/month > 600 Euro/month	13 (44.8) 7 (14.9)	16 (55.2) 40 (85.1)	< 0.01
Years with T1DM, median (25%–75%)		14.0 (12.0 – 17.8)	14.0 (9.0 – 23.0)	0.73
T1DM expenses	≤ 100 Euro/month > 100 Euro-month	1 (2.1) 18 (48.6)	46 (97.9) 19 (51.4)	< 0.01
Hypoglycemia — week, median (25%–75%)		3.0 (2.0 – 5.0)	3.0 (1.0 – 5.0)	0.52
Hypoglycemia — half-year, median (25%–75%)		1.2 (0.0 – 32.7)	0.0 (0.0 – 6.0)	0.16
Number of HbA1c tests in 2020, N (%)	0 1 2 3 4	3 (50.0) 9 (24.3) 4 (19.0) 2 (18.2) 2 (16.7)	3 (50.0) 28 (75.7) 17 (81.0) 9 (81.8) 10 (83.3)	0.54
HbA1c at the last medical check, mean ± SD		11.1 ± 13.6	7.7 ± 2.2	0.20
HbA1c < 7, N (%)	< 7 ≥ 7	5 (14.7) 14 (27.5)	29 (85.3) 37 (72.5)	0.19

### Quality of life and self-management

The reliability of all scales was high, ranging from α = 0.75 to α = 94 for all blocks for both questionnaires (excluding the SM Diet that had medial reliability; α = 0.63). Correlation between QoL and self-management was weak and partly insignificant. Self-management blocks correlated among themselves significantly, but not strongly (Additional file 1: Table S1). No significant relations were found between the number of tests and three self-management blocks (*p* = 0.12, *p* = 0.86, and *p* = 0.36, respectively).

Significant differences at the 0.1 significance level were observed between user groups in their Therapy and Concerns blocks, and in Total QoL. The highest values for both groups were found for Therapy and Communication blocks of QoL. There were no significant differences between groups in their self-management blocks (Table 2). Univariate relationships were found between Total QoL and sex (*p* = 0.03).

**Table 2:** Quality of life and self-management, grouped by method of insulin administration

Variable, median (25%–75%)	Insulin Pump Users, N = 20	Multiple Daily Injection Users, N = 67	*p *value
Signs and symptoms	63.3 (52.5–77.9)	61.7 (48.3–73.3)	0.55
Therapy	87.5 (66.3–95.0)	75.0 (60.0–85.0)	0.07
Care	79.2 (59.4–91.7)	70.8 (58.3–83.3)	0.31
Concerns	62.5 (43.8–75.0)	50.0 (25.0–62.5)	0.02
Communication	89.6 (71.9–99.0)	79.2 (62.5–95.8)	0.23
Total QoL	72.7 (64.1–86.7)	66.0 (51.8–79.0)	0.09
General	59.0 (54.8–69.8)	66.0 (47.0–81.0)	0.34
Diet	37.5 (30.0–49.4)	45.0 (30.0–52.5)	0.80
Physical activities	60.0 (37.9–78.8)	48.3 (35.0–70.0)	0.29
Total SM	49.5 (43.9–62.9)	52.9 (39.8–61.3)	0.98

In fully adjusted multiple regression models, pump users were seven times more likely to have a high Total QoL than injection users (OR 7.38; CI 0.87; 62.9). Factors that increased Total QoL were lower age, male sex, lower T1DM expenses (the most prominent association), more years living with T1DM, and better self-management. Most of the confidence intervals were wide, pointing to the low number of participants in the study (Table 3). However, the post hoc calculated power of analysis was 70.1% (*p* = 0.01), indicating the study’s medial power.

**Table 3:** Association between Total QoL and demographic and T1DM-related factors

Variable	Odds ratio, OR	95% confidence interval (CI)	*p* value
Method of insulin administration	7.38	0.87; 62.9	0.07
Age	0.90	0.82; 0.99	0.02
Sex	9.62	2.20; 42.1	< 0.01
Education	0.26	0.06; 1.06	0.07
Income	0.63	0.16; 2.66	0.53
T1DM expenses	7.01	1.29; 38.0	0.02
Years with T1DM	1.16	1.05; 1.30	< 0.01
Total SM	1.07	1.02; 1.13	< 0.01

### Additional and sensitivity analyses

For pump users, the main reason to use a pump was improved QoL; this was mentioned by 90% of them. For injection users, the median time they had been using insulin injections was eight years, and the main reason for not using a pump was its cost, as mentioned by almost half of these respondents. Of the 13 patients that previously used a pump, the main reason why they stopped was the cost (mentioned by 46.2% of those that stopped using a pump) (Additional file 1: Table S2).

In the univariate analysis between the Therapy block of QoL and demographic and T1DM-related factors, significant relationships at the 0.1 significance level were found for years with T1DM (*p* < 0.01) and T1DM expenses (*p* = 0.08); for the Communication block, significant univariate relationships were found for the number of hypoglycemic episodes per week (*p* = 0.09) and sex (*p* = 0.07). Consistent with the main analysis, male sex, lower T1DM expenses, and years living with T1DM were associated with better Therapy and Communication blocks (Additional file 1: Table S3).

### Qualitative part of the study

Of those included in the quantitative part of the study, 8 pump users and 13 injection users also participated in the qualitative interviews; 15 of these were women. The men-women proportion in each study arm was similar to that in the quantitative part of the study.

The age of the interviewees ranged from 18 to 50 years, and years with T1DM ranged from 1 to 35. Eight participants did not have T1DM diaries, three had one only at the beginning of their treatment, two use them only for visits with a physician, and six regularly recode their activities in their diaries (two using an app to do so). One participant kept a diary when she used multiple insulin injections but stopped when she switched to an insulin pump (Table 4).

**Table 4:** Main characteristics of interviewed participants

Number of the interview	Name in the study*	Age range	Age when diabetes was diagnosed	Years with T1DM	Having T1DM diary	Method of insulin administration
1	E	20–30	10	13	No	Multiple daily injections
2	B	20–30	26	1	No	Multiple daily injections
3	F	50–60	19	31	No	Multiple daily injections
4	D	20–30	12	12	At the beginning	Insulin pump
5	L	10–20	9	9	Till started to use a pump	Insulin pump
6	M	10–20	10	8	For physician visits only	Insulin pump
7	C	20–30	16	9	Yes	Insulin pump
8	G	20–30	9	16	At the beginning	Insulin pump
9	R	30–40	27	11	At the beginning	Multiple daily injections
10	O	30–40	12	25	Yes	Multiple daily injections
11	N	30–40	24	20	Yes	Multiple daily injections
12	Z	30–40	15	18	No	Multiple daily injections
13	I	30–40	28	7	App	Multiple daily injections
14	V	30–40	2.5	34.5	No	Multiple daily injections
15	S	40–50	8	35	No	Multiple daily injections
16	J	30–40	26	5	Yes	Multiple daily injections
17	Q	20–30	8	18	For physician visits only	Multiple daily injections
18	K	30–40	17	21	App	Multiple daily injections
19	T	30–40	17	19	App	Insulin pump
20	P	20–30	4	18	No	Insulin pump
21	A	20–30	9	11	No	Insulin pump

^*^No relation between the name in the study and the participant’s real name

Analysis of 40 identified codes of the interviews revealed three major themes of answers: diagnosis-related, daily self-management, and life with T1DM. Each of the major themes was further divided into three to four subcategories (Table 5). Here we will present a part of the results related to one subcategory for each category of answers: perception of diagnosis (major theme: diagnosis-related), insulin administration (major theme: daily self-control), and T1DM-related costs (major theme: life with T1DM).

**Table 5:** Categories of answers of interview participants

Major theme	Subcategory
Diagnosis-related	Perception of diagnosis
	T1DM-related trainings in different ages
	Coming back to feel in studies and at work
Daily self-control	Insulin administration
	Measure of the level of glucose
	Nutrition and physical activity
	T1DM diary
Life with T1DM	T1DM-related costs
	Pregnancy
	Need for additional support

#### Perception of diagnosis

Before their diagnosis, most participants had had some symptoms that they had not related to T1DM, such as thirst, frequent urination, weight loss, and weakness. Therefore, for nearly all of them, the diagnosis was unexpected and shocking. For example, I, who was diagnosed at the age of 28 after being hospitalized due to T1DM:

*I didn't know anything before, it seemed to me that diabetes could be born or not. I was so bad in that resuscitation because I was in a severe hypoglycemic condition … my head was dull … it was so hard to grasp.*

This reaction was not related to the participant’s age at the time of diagnosis (Additional file 1: Supplement 5).

#### Insulin administration

One of the main reasons to use an insulin pump was the QoL that it provides (Additional file 1: Supplement 6). For example, M said:

*I have much more control with the pump, because I can adjust insulin doses if necessary, and adjust the time for basal insulin. I can stop insulin if needed. with the syringe, you are injecting and then you can no longer control what is going. The pump gives much more control to both the doctor and the patient, if a person understands how the pump works. But that's what training is for.*

However, some of the injection users saw positive aspects in their treatment method as well. For example, I, who uses the injections:

*When using injections, it is nice to inject insulin once and that is*.

F, who has used injections for 31 years, was categorically opposed to the idea of a pump:

*No, never! It is not practical for me to have a foreign object that is always present at my waist area. I feel very uncomfortable. That limits me*.

To summarize: although QoL was mentioned by most of the participants as the determining factor for use of the pump, some participants feel that a pump is less comfortable and even disturbing. This supports the quantitative result showing a lack of proper relations between the method of administration and QoL.

#### T1DM-related costs

Most pump users in our study mentioned the cost of this administration method (Additional file 1: Supplement 7). For some participants, the decision whether to use a pump depends on the monthly costs. For example, K said:

*It is an extra investment* [talking about the pump]*—now I have needles and insulin for free, I do not have to buy anything extra—just those test strips, because the glucometer is also free for me. Together it's pretty affordable*.

D switched from the pump to injections several times because of financial problems:

*I had already used it [pump] as a child, I was 13 years old. […] I used to have insulin pens, but then my mom saved money so I could have the pump. […] After that I had to switch back to insulin pens because I was in big financial trouble. However, I really wanted to get back to the pump.*

In Latvia, state reimbursement for insulin pumps is possible until the age of 18. Thus, some people are forced to switch to injections at that point. For most of the participants who would like to use an insulin pump, treatment-related costs are too high, and some of them were forced to change to the cheaper injection method. This supports the quantitative result of the study on the relation between T1DM-related costs and QoL.

## Discussion and conclusions

In this study, we investigated quantitatively and qualitatively factors related to the QoL of patients with T1DM according to their method of insulin administration: using an insulin pump or using multiple daily injections. The reported QoL was found to be associated with the method of insulin administration, the age and sex of the participants, the number of years the patient had lived with T1DM, self-management, and T1DM-related expenses. QoL was the main reason cited for using a pump, while the expense was the main reason to avoid its use or to stop using it.

An association between the method of insulin administration and the QoL of patients with T1DM has been shown previously both in qualitative and in quantitative studies [11]. However, until recently, most of the studies on insulin pumps were qualitative and were performed on populations of children [26–28]. In the last decade, quantitative evaluations of pump use had appeared as well, but studies combining these two methods of investigation are still scarce. However, similarity among their objectives allows us to combine the results of different studies to provide additional explanations of our observed results. For example, Alqambar et al. found higher scores for QoL for pump users than for injection users. The former had significantly higher satisfaction with their treatment and had a lower burden of disease (both with *p* < 0.01) [29]. These results are supported by the qualitative study by Mesbah et al., which described higher satisfaction among pump users in many areas [22]. In our study, although we did not observe statistically significant differences in QoL between pump and injections users, QoL was the main reason given for using the pump. Nevertheless, in our study some participants had a negative attitude toward the pump. Mesbah et al. likewise report the existence of negative feelings toward pumps, such as fear of being dependent on a machine or concern about sporadic mechanical problems [30].

As QoL is multidimensional, factors affecting it might differ according to study design and measures. For example, in our study we did not observe any association of QoL with the level of HbA1c. In contrast, in the study by Alavrado-Martel et al. performed in Spain, worse QoL was associated with increasing HbA1c [31]. This fact is extremely interesting, as in both studies the mean age of participants was 31 years and mean years living with T1DM were 14, and participants had similar levels of education. It is possible that the difference can in part be explained by the proportion of pump users: a third of our participants use a pump and therefore are in reduced risk of an increased level of blood sugar, but in the Spanish study only 5% of patients were pump users. Therefore, the association with the level of HbA1c was not prominent. In addition, in the Spanish study a better QoL was associated with the female sex, but in our study, it was associated with the male sex. As mentioned by Mesbah et al., lack of flexibility in clothing options can reduce the QoL of female pump users [30], and this may be reflected in our results. In another study [32] women with diabetes were found to evaluate their health status and diabetes-related care worse than men; they also had more diabetes-related worries related to higher levels of Hb1Ac, although their level of metabolic control did not differ from that of men.

Initiation of pump therapy in Latvia usually is not a choice, but a costly necessity due to problems in diabetes management (such as hypoglycemia) and discomfort associated with diabetes treatment (e.g., pain, fear of injections). Studies describe substantial clinical benefits of insulin pumps for such patients. For example, in the meta-analysis by Benkhadra et al. based on 25 randomized clinical trials, absolute HbA1c reduction was better managed in pump users than in injection users (difference of 37%, CI 0.24; 0.51), and this result was consistent across adults and children. In addition, pump users had a lower risk of hypoglycemia (relative risk, RR = 0.85, CI 0.6–1.2) [10]. These results were supported by another meta-analysis by Jeitler et al. that analyzed 33 studies and found a 43% difference (CI – 0.65; – 0.20) between groups with different methods of insulin administration [8]. In addition to clinical benefits, the patient’s ability to self-manage should be considered when choosing the method of administration. Our study did not observe any difference in self-management between pump users and injection users, but we do observe a slight but significant increase in QoL for those with better self-management. The main cause seems to be educational training provided for all patients with T1DM. Previous studies have described the effectiveness of such training on self-management. For example, in the structural analysis by Campbell et al. based on 18 studies, people who attended educational training gained clinical benefits by managing their lives according to the knowledge they received during these sessions. However, people were often tired and encountered difficulties in managing their everyday lives according to guidelines even during these educational trainings, which made additional follow-up by the physician essential [7]. For pump users less intensive follow-up is needed, thus removing a level of stress from both physician and patient. Overall, a personal approach when choosing the method of insulin administration seems to be the best in the case of T1DM patients.

### Limitations of the study

The main limitation of our study is its cross-sectional nature, which does not allow us to evaluate causal relationships. The study included a small number of participants, especially in the pump-users group, and we did not divide participants into age groups. Further, the use of the Internet for enrollment limited the available pool of participants and may introduce a volunteer bias that can affect the validity of the results. Further limitations include self-evaluation of QoL and self-management and possible errors regarding the number of medical checks due to memory bias. Specific questions on installation, operation, troubleshooting, and handling of the insulin pumps (these factors could affect the quality of therapy in pump-users and have an impact on QoL) were not included in the study to avoid complexity. In addition, some limitations in the qualitative part of the study could be related to the language, as the native language of the interviewer was Latvian. Despite the good knowledge of Russian, some impreciseness could occur.

Although the reliability of all parts of the survey was high at the initial stage of their check, we observed the medial reliability of one of its parts after collecting the information about all study participants. As we did not see any difference between the insulin pump users and multiple injection users in other parts of the SM questionnaire, we assumed that the lower reliability of this part of the questionnaire will not affect the results of our study.

Another limitation of our study is the high proportion of insulin pump users. Before we start the study, we knew that the number of insulin pump users in the Latvian population is relatively small. Therefore, we decided to invite participants in the proportion of 1:3 (pump users *versus* injection users) to increase the overall power of analysis. We attempt to invite as many pump users as it was feasible. As a result of this strategy, we observed a disproportion between the pump users and injection users in their relation to the whole pump/injection users’ population in the country. This can affect the results of our study, especially the qualitative part of them.

### Strength of the study

A major strength of our study is a mixed methodology that allows us to describe QoL-related parameters of T1DM patients from various sides. Further, although the sample size was small, it represented more than half of all pump users in Latvia, and regardless of the small sample size, the power of analysis was 70%.

## Conclusions

QoL was the main reason to use an insulin pump, while the main reasons to avoid one were expenses related to its use. However, the expense related to diabetes treatment, not the method of insulin administration, was the strongest predictor of T1DM patients’ QoL. Reimbursement policies thus should not only consider the patient’s personal preference for treatment, but also be structured to alleviate ongoing maintenance costs, particularly as high costs drive reduced adherence to treatment regimens that in turn impose higher costs on the healthcare system in the form of additional disorders and comorbidities.

Development of national insurance policies is critical worldwide, but especially in countries like Latvia with overall weak health care and public health systems, supporting reimbursement for insulin pumps could help:

1. to reduce complications related to poor treatment adherence,
2. to avoid increased additional morbidity, and
3. to prevent an overload of the health system.

## Supplementary Information

**Additional file 1**. Surveys by blocks, qualitative responses of participants, supplemental tables and figures.

## Data Availability

The datasets generated and/or analyzed during the current study are available in the Longenesis Curator platform, accessible via following link: www.longenesis.com/curator.

## Abbreviations

T1DM: Type I diabetes mellitus
HbA1c: Glycated hemoglobin
QoL: Quality of life
OR: Odds ratio
CI: 95% Confidence interval
RR: Relative risk
