# Supplementary material for: Quantitative and qualitative analysis of the quality of life of Type 1 diabetes patients using insulin pumps and of those receiving multiple daily insulin injections
Source: Health Qual Life Outcomes. 2022 Aug 1;20:120. doi: 10.1186/s12955-022-02029-2 (PMC9344781; doi:10.1186/s12955-022-02029-2)
Supplement: Supplementary file 1 — Additional file 1. Surveys by blocks, qualitative responses of participants, supplemental tables and figures. [file 12955_2022_2029_MOESM1_ESM.docx]

**Supplemental materials**

Supplement 1. Socio-demographic data survey

1. What is your age?

2. What is your gender?

3. Are you pregnant now?

4. What is your weight (kg)?

5. What is your height (cm)?

6. What is your education?

7. What are your living conditions?

8. What is your household income per person per month (EUR)?

9. What is your monthly expenditure on diabetes care (EUR)?

10. To what extent does your financial situation meet your needs?

Supplement 2. Factors related to T1DM

11. In what year were you diagnosed with diabetes?

12. What is your current insulin delivery method?

13. Have you used a pump before?

14. Why did you stop using your insulin pump?

15. How many times a day do you usually inject insulin?

16. How many years have you been using an insulin pump?

17. What insulin pump/pump are you currently using?

18. What are the main reasons you use an insulin pump?

19. Are you using a continuous glucose monitoring system?

20. Why not use a continuous glucose monitoring system?

21. How many days a month do you use the continuous glucose monitoring system?

22. What continuous glucose monitoring system do you use most often?

23. Please enter your latest glycosylated haemoglobin HbA1c laboratory results (%)

24. How many units of insulin do you consume on average per day (U/d)?

25. How many times a week do you usually have a blood glucose level below 4 mmol/l, have symptoms of hypoglycaemia and are you dealing with it yourself?

26. How many times in six months do you usually have a blood glucose level below 4 mmol/l, have symptoms of hypoglycaemia and need help from peers?

Supplement 3. QOL questions by blocks

*QOL block 1: Diabetes signs and symptoms*

1. I feel hungry

2. I feel thirsty

3. I have to go to the bathroom too often

4. I have stomach aches

5. I have headaches

6. I feel like I need to throw up

7. I go “low”

8. I go “high”

9. I feel tired

10. I get shaky

11. I get sweaty

12. I feel dizzy

13. I feel weak

14. I have trouble sleeping

15. I get cranky or grumpy

*QOL block 2: Diabetes therapy*

1. It hurts to get my finger pricked

2. It hurts to get insulin shots

3. I am embarrassed by my diabetes treatment

4. My spouse, significant other, and/or other family members and I argue about my diabetes care

5. It is hard for me to do everything I need to do to care for my diabetes

*QOL block 3: Diabetes care*

1. It is hard for me to take blood glucose tests

2. It is hard for me to take insulin shots

3. It is hard for me to exercise

4. It is hard for me to keep track of carbohydrates

5. It is hard for me to carry a fast-acting carbohydrate

6. It is hard for me to snack when I go “low”

*QOL block 4: Diabetes concerns*

1. I worry about going “low”

2. I worry about going “high”

3. I worry about long-term complications from diabetes

4. Daily life with diabetes causes me added stress

*QOL block 5: Diabetes communication*

1. It is hard for me to tell the doctors and nurses how I feel

2. It is hard for me to ask the doctors and nurses questions

3. It is hard for me to explain my illness to other people

4. I am embarrassed about having diabetes

5. Having diabetes limits my sex life

6. I feel limited in my professional life because of diabetes

Supplement 4. Self-management questionnaire by block

*SM block 1: General*

1. I check my blood sugar levels with care and attention

2. I keep all doctors’ appointments recommended for my diabetes treatment

3. I take my diabetes medication as prescribed

4. I record my blood sugar levels regularly (or analyse the value chart with my blood glucose meter)

5. I tend to avoid diabetes-related doctors’ appointments

6. I tend to forget to take or skip my diabetes medication

7. I do not check my blood sugar levels frequently enough as would be required for achieving good blood glucose control

8. Regarding my diabetes care, I should see my medical practitioner(s) more often.

9. My diabetes self-care is poor

10. I feel satisfied with my daily involvement in the self-care of my diabetes

*SM block 2: Diet*

1. Occasionally I eat lots of sweets or other foods rich in carbohydrates

2. The food I choose to eat makes it easy to achieve optimal blood sugar levels

3. I strictly follow the dietary recommendations given by my doctor or diabetes specialist

4. Sometimes I have real ‘food binges’ (not triggered by hypoglycaemia)

5. Despite diabetes, I feel free and can choose my diet flexibly

*SM block 3: Physical activities*

1. I do regular physical activity to achieve optimal blood sugar levels

2. The food I choose to eat makes it easy to achieve optimal blood sugar levels

3. I tend to avoid diabetes-related doctors’ appointments

4. I feel satisfied with the time I spend on physical activity

5. I have a good rest and my sleep is good

6. I can enjoy physical activity whenever I want

Supplement 5. Perception of T1DM diagnosis

For example, L:

*At the time ... well, I was only about 9 years old, and my mom and I were together [in the hospital]. We just cried the first days all the time. It was terrible ... Yes, the first day in the hospital, when we realized what and how [crying] ...*

Likewise, G, who also was diagnosed approximately about the age of 9, mentions the shock to a family:

*I may have heard minimally about diabetes. Yes, and the family took it very seriously, Dad even wanted to commit suicide ... However, despite everything, he had to adjust quite quickly, understand how to act, otherwise it would not be possible to live on.*

However, Z, who was diagnosed approximately at the age of 15, remembers that a family had more experience, and S, who was diagnosed approximately at the age of 8, mentioned that tried to approach the diagnosis with a cool mind and peace in her large family. These initial feelings were the starting points from which participants began their T1DM-related therapy.

Supplement 6. Insulin administration

On the other hand, some injection users mentioned the possibility of forgetting the injection or the insulin itself and therefore being forced to change their plans. For example, Z once had to return to get an insulin after starting on his way to Poland. E describes such a situation:

*Of course, these cases have been different. It happened that we didn't notice that the insulin ran out — we were visiting friends — I lived without insulin for one day and had to go to the hospital.*

Supplement 7. T1DM-related costs

A, who uses an insulin pump, said:

*I receive a disability benefit — a disabled person since childhood, which is about 126 EUR per month, but it does not cover the full cost of diabetes per month — 150–200 EUR. And now the insulin I use was taxed... It is not like I am in poverty, I can still afford it, but without parental support and having to live alone... It would be completely unrealistic for a student. Then you would have to think of another scenario — either your studies would fail, and you would have to look for a job....*

This is also pointed out by P, whose parents bought a pump:

*Yes, the parents bought it themselves and in fact it was a very large sum of money. It was expensive, but how much, I do not remember. Everything was covered by parents and grandparents. Also, now I cover the cost of the pump myself. [...] I think that diabetes should not be included in a disability group 3, but it should be a separate group — 120 EUR per month is not enough.*

T also does not hide the cost:

*We bought the pump ourselves for the full amount and, frankly, the device costs like a car. And the monthly cost is also impressive. There are countries where these costs are fully reimbursed.*

L and M, both in their 18s and still receiving an insulin pump maintenance allowance, are worried about what will happen after they finish school (12th grade), knowing that they will have to switch to injections. L commented:

*It would be nice if we did not have to switch back, because that's our daily routine! We did not choose to be sick or not. Yes, now I'm in my 18s — and what's the difference that I was 17 the day before and 18 the next?*

Supplement Table 1. Reliability of questionnaires and correlations between QOL and SM

| Scale | Alpha Cronbach | Therapy | Care | Concerns | Communication | General | Diet | Physical activities | SM total |
| --- | --- | --- | --- | --- | --- | --- | --- | --- | --- |
| Signs and symptoms | 0.87 | 0.59** | 0.68** | 0.61** | 0.67** | 0.07 | 0.25* | 0.38** | 0.32** |
| Therapy | 0.76 |  | 0.71** | 0.68** | 0.71** | 0.21 | 0.39** | 0.40** | 0.45** |
| Care | 0.75 |  |  | 0.67** | 0.68** | 0.29** | 0.38** | 0.49** | 0.54** |
| Concerns | 0.78 |  |  |  | 0.62** | -0.05 | 0.73 | 0.26* | 0.13 |
| Communication | 0.86 |  |  |  |  | 0.35** | 0.32** | 0.36** | 0.47** |
| QOL Total | 0.94 |  |  |  |  | 0.20 | 0.32** | 0.43** | 0.44** |
| General | 0.85 |  |  |  |  |  | 0.49** | 0.23** | 0.77** |
| Diet | 0.63 |  |  |  |  |  |  | 0.24** | 0.74** |
| Physical activities | 0.77 |  |  |  |  |  |  |  | 0.70** |

**significant at 0.05 level

Supplement Table 2. Specific information of IPU and MDIU

| Variable | Categories |  |
| --- | --- | --- |
| IPU, N = 20 | | |
| Pump-use years,  mean ± SD |  | 7.2 ± 4.7 |
| Reasons to use pump | Quality of life  Insulin dosing  Less pain  Less hypoglycemia  Trying  Other | 18 (90.0)  15 (75.0)  9 (45.0)  7 (35.0)  2 (10.0)  3 (15.0) |
| MDIU, N = 67 | | |
| Insulin time/ day, mean ± SD |  | 4.1 ± 1.2 |
| Reasons for not using a pump | Expensive  Information  Do not want  No model  Do not trust  Other | 33 (49.3)  13 (19.4)  8 (11.9)  5 (7.5)  3 (4.5)  5 (7.5) |
| Previous pump use, N (%) | Yes | 13 (19.4) |
| Reasons to stop using pump, N (%) | Expensive  Not comfortable  Do not trust  No result  Other | 6 (46.2)  4 (30.8)  1 (7.7)  2 (15.4)  5 (38.5) |

Supplement Table 3. Association between Therapy and Communication blocks of QOL and demographic and T1DM-related factors

|  | Therapy block of QOL | | | Communication blocks of QOL | | |
| --- | --- | --- | --- | --- | --- | --- |
| Variable | Odds ratio, OR | 95% CI | *p* value | Odds ratio, OR | 95% CI | *p* value |
| Method of insulin administration | 3.13 | 0.48; 20.5 | 0.23 | 3.11 | 0.52; 18.56 | 0.21 |
| Age | 0.93 | 0.85; 1.03 | 0.15 | 0.90 | 0.83; 0.98 | 0.02 |
| Sex | 5.04 | 1.30; 19.5 | 0.02 | 3.06 | 1.00; 9.31 | 0.05 |
| Education | 0.98 | 0.25; 3.79 | 0.98 | 0.73 | 0.22; 2.37 | 0.56 |
| Income | 1.76 | 0.42; 7.35 | 0.44 | 0.60 | 0.17; 2.15 | 0.43 |
| T1DM expenses | 0.91 | 0.21; 4.04 | 0.91 | 10.05 | 1.99; 50.9 | < 0.01 |
| Years with T1DM | 1.17 | 1.06; 1.30 | < 0.01 | 1.11 | 1.03; 1.21 | 0.01 |
| SM total | 1.06 | 1.01; 1.11 | 0.02 | 1.05 | 1.00; 1.10 | 0.03 |


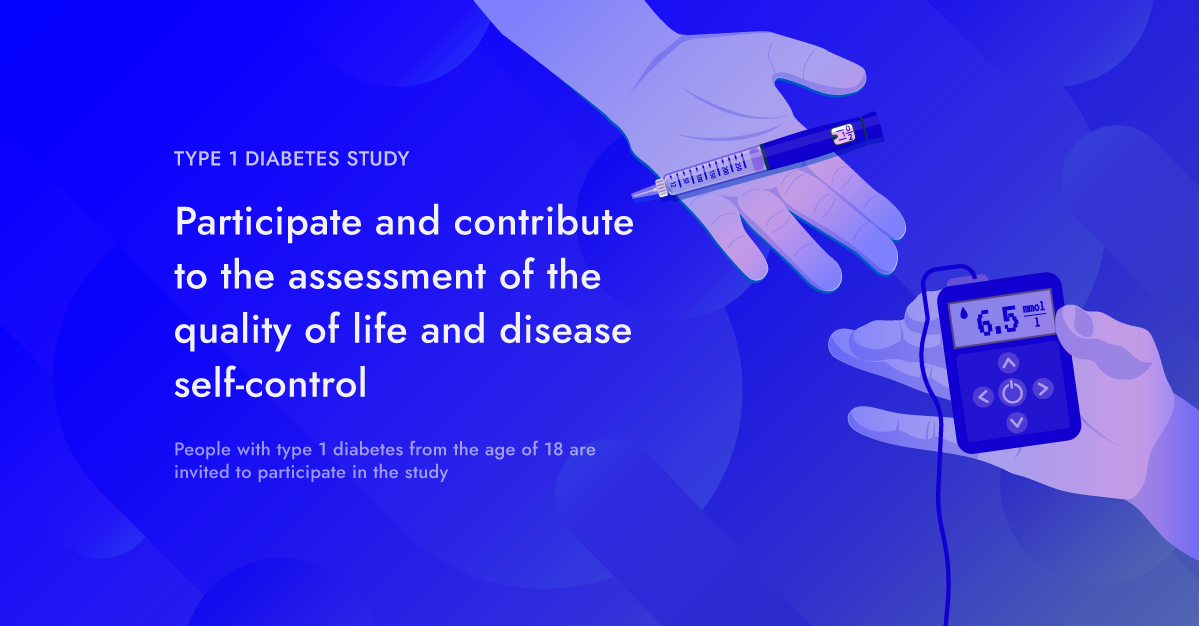


Supplement Figure 1. Visual engagement material used in study invitations


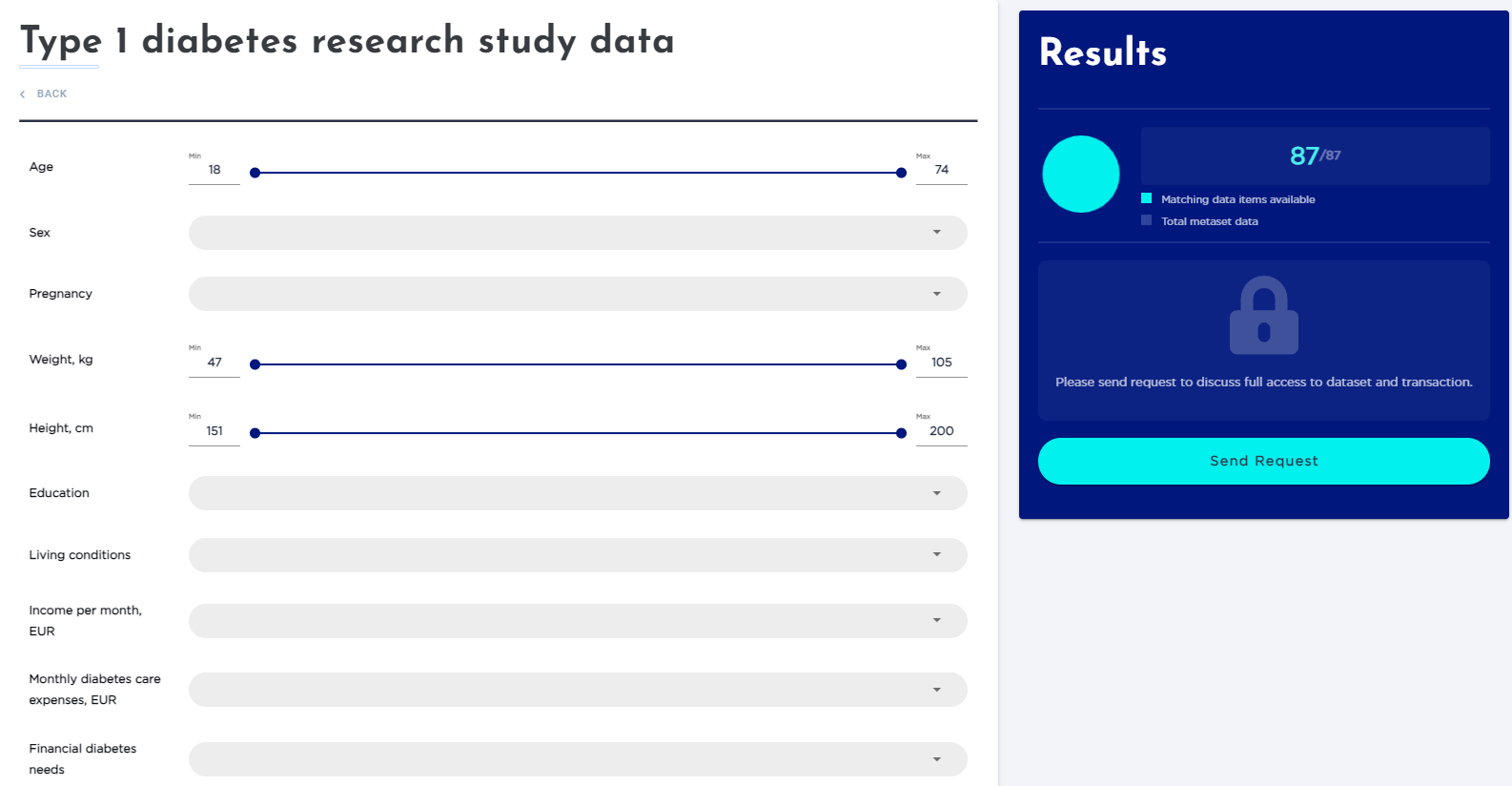


Supplement Figure 2. Electronic platform – research study metadata query view
